# Preliminary research on tailored fluid therapy in pigs: comparing customized ionic solutions with Hartmann’s solution

**DOI:** 10.1186/s12917-024-04145-1

**Published:** 2024-06-26

**Authors:** Seongju Lee, Seung-Eun Lee, Jae-Ik Han, Sang Chul Lee, Yubyeol Jeon

**Affiliations:** 1 Department of Theriogenology and Reproductive Biotechnology, College of Veterinary Medicine, Jeonbuk National University, Iksan, 54596 Republic of Korea; 2 Cronex Inc., Cheongju, 28174 Republic of Korea; 3 Subtropical Livestock Research Institute, National Institute of Animal Science, RDA, Jeju, 63242 Republic of Korea; 4 Laboratory of Wildlife Medicine, College of Veterinary Medicine, Jeonbuk National University, Iksan, 54596 Republic of Korea

**Keywords:** Fluid therapy, Ionic concentration, Pig physiology, Hartmann’s solution, Customized fluid, Clinical trials

## Abstract

**Background:**

Fluid therapy in veterinary medicine is pivotal for treating various conditions in pigs; however, standard solutions, such as Hartmann’s solution, may not optimally align with pig physiology. This study explored the development and efficacy of a customized fluid therapy tailored to the ionic concentrations of pig blood, aiming to enhance treatment outcomes and safety in both healthy and diseased pigs.

**Results:**

The study involved two experiments: the first to assess the safety and stability of customized fluids in healthy pigs, and the second to evaluate the efficacy in pigs with clinical symptoms of dehydration. In healthy pigs, the administration of customized fluids showed no adverse effects, with slight alterations observed in pO2, hematocrit, and glucose levels in some groups. In symptomatic pigs, the customized fluid group did not show any improvement in clinical symptoms, with no significant changes in blood chemistry or metabolite levels compared to controls. The customized fluid group showed a mild increase in some values after administration, yet within normal physiological ranges. The study reported no significant improvements in clinical or dehydration status, attributing the observed variations in blood test results to the limited sample size and anaesthesia effects rather than fluid characteristics.

**Conclusions:**

Customized fluid therapy, tailored to mimic the ionic concentrations of pig blood, appears to be a safe and potentially more effective alternative to conventional solutions such as Hartmann’s solution for treating pigs under various health conditions. Further research with larger sample sizes and controlled conditions is recommended to validate these findings and to explore the full potential of customized fluid therapy in veterinary practice.

## Background

The significance of fluid therapy in veterinary medicine, specifically concerning preserving the health and welfare of pigs, cannot be emphasized enough. In veterinary medicine, fluid therapy is an important treatment for small animals. It is used for multiple purposes such as correcting dehydration, maintaining proper blood volume, addressing electrolyte imbalances, and ensuring proper transport of fluids within the body [1].

Pigs are mostly administered oral electrolyte solutions to treat diarrhea caused by *Escherichia coli*, rotavirus [2], and transmissible gastroenteritis virus [3]. Nevertheless, sows, particularly those that are pregnant, in labor, or postpartum, are commonly treated with intravenous (IV) fluid. In such cases, 5% dextrose IV is administered to treat sows with dystocia caused by uterine inertia [4]. Furthermore, IV fluid therapy is essential for maintaining hydration, supporting cardiovascular function, correcting electrolyte imbalances, preventing complications, and ensuring experimental consistency in pigs undergoing anesthesia in research settings [5]. Proper fluid therapy protocols should be established and followed to optimize the safety and well-being of the animals and to obtain reliable research results. Inadequate fluid therapy during anesthesia can lead to complications such as acute kidney injury, hypovolemic shock, and impaired tissue oxygenation [6, 7]. Therefore, it is crucial to understand the physiological requirements of pigs and to tailor fluid therapy accordingly to ensure the best possible outcomes in both experimental and clinical settings.

IV fluids can be categorized into colloidal and crystalloid solutions. Owing to their higher molecular weight, colloids are more efficient in expanding the intravascular compartment. Crystalloids are aqueous solutions containing both inorganic and small organic compounds. Crystalloids that have an ionic composition similar to plasma may be described as “balanced” or “physiological“ [8]. Hartmann’s solution (H/S) is a type of isotonic crystalloid that is similar to lactated Ringer’s solution and is commonly given to animals during surgery [9]. The solution contains sodium chloride, sodium lactate, potassium chloride, and calcium chloride dissolved in water, which closely resembles the ionic composition of blood plasma [10]. The osmolarity of this fluid is comparable to that of extracellular fluid, which means that about 20% of the administered volume will remain in the intravascular space [11]. It is suitable for fluid resuscitation in patients with metabolic acidosis, as it can enhance perfusion and restore circulating blood volume. Small animal veterinarians typically choose isotonic balanced crystalloid solutions over other commonly used options such as isotonic saline (0.9% NaCl) solution for IV fluid treatment [12]. Although H/S is commonly employed as the standard solution in calves with mild dehydration [13], this formulation is not optimum for ruminants and is generally insufficient for treating neonatal calves or adult cattle with severe acidemia or alkalemia, hyponatremia, hypokalemia, or hypochloremia [14]. Indeed, the composition of authentic pig serum varies [15] necessitating the development of a corresponding fluid.

Point-of-care testing (POCT) has become more common in veterinary practices recently [16], especially for large animals compared to companion animals [17]. The validity of the enterprise point-of-care (EPOC) blood analysis system ( Epocal Inc., ON, Canada), a portable blood analyzer, has been demonstrated in several animal species [18, 19]. Previously, blood analysis in pigs predominantly relied on conventional laboratory techniques [20] or alternative point-of-care (POC) blood analyzers [21], with the exclusion of EPOC utilization. There has been limited research involving the application of EPOC for this specific purpose.

This study aims to introduce the creation and implementation of personalized fluid treatments for pigs, designed to precisely mimic the ionic concentrations observed in pig blood. This novel strategy seeks to improve the effectiveness and safety of fluid therapy in veterinary medicine, specifically in swine. In addition, the purpose of this study is to enhance treatment outcomes in different clinical situations, such as dehydration and disease recovery, by precisely modifying fluid composition according to the specific physiological needs of pigs. The present study makes a substantial contribution to veterinary practice by providing a more focused and efficient therapeutic approach for controlling fluid therapy in pigs.

## Results

### Quantitative composition of the customized fluids

Target concentrations (Table 1) of each ion were determined using species-specific serum chemistry reference intervals [20, 22, 23]. All components of the customized fluid were purchased from Sigma-Aldrich (St. Louis, MO, USA).

**Table 1:** Compositions of customized fluids for each group

Group	Target Ion Concentrations (mEq/L)					Component Amounts (g/L)				Osmolarity(mOsm/L)
	Na^+^	K^+^	Ca^2+^	Cl^−^	Lactate^−^	NaCl	KCl	CaCl2	Sodium Lactate	
Hartmann’s solution	130	4	2.7	109	28	5.0	0.30	0.20	3.10	278
Group 1	150	3.9	2.6	109	30	5.0	0.30	0.20	5.90	316
Group 2	150	3.9	2.6	118	28	6.2	0.30	0.20	3.10	327
Group 3	130	6	2.7	115	28	5.0	0.45	0.20	3.10	319
Group 4	150	6	2.6	123	30	5.6	0.45	0.20	5.90	346

Ion concentrations are expressed in milliequivalents per liter (mEq/L). The amount of each component is expressed in grams per liter (g/L). The osmolarity is determined via calculation

The amount of each component was adjusted to match the target ion concentration (Table 1). In addition, osmolarity measurements were conducted because of the increased quantity of the respective chemicals in H/S.

### Evaluating the stability of customized fluid by injecting it in healthy pigs

**Fig. 1:**
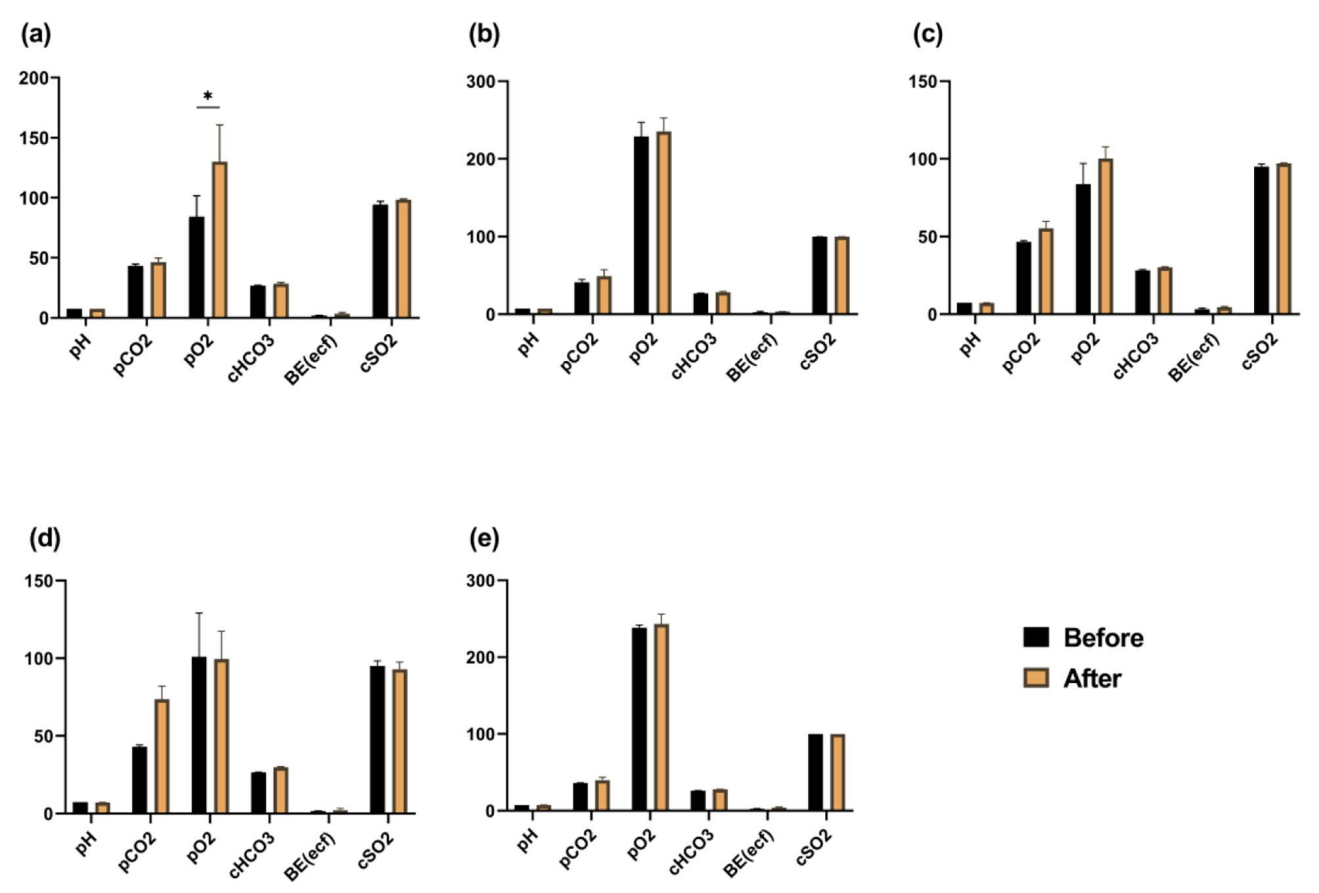
Blood gas analysis results before and after fluid administration in Experiment 1. Mean values and standard error of the mean (SEM) for each group are presented. The asterisk (*) denote statistically significant differences (*p* < 0.05) between pre- and post-fluid administration values. (**a**): Group1; (**b**): Group2; (**c**) Group3; (**d**) Group4; and (**e**) Control (H/S). (pCO2, pO2 = mmHg; cHCO3^−^, BE (ecf) = mmol/L; cSO2 = %)

We performed a comparative analysis of blood test results by assessing the mean values of blood parameters before and after the administration of tailored fluids to healthy pigs. Šídák’s post-hoc multiple comparison tests were applied following a 2-way ANOVA to identify any discrepancies. Regarding blood gases, the only alteration observed before and after fluid administration was in the pO2 level of group 1, which received a higher dosage of sodium lactate (Fig. 1).

**Fig. 2:**
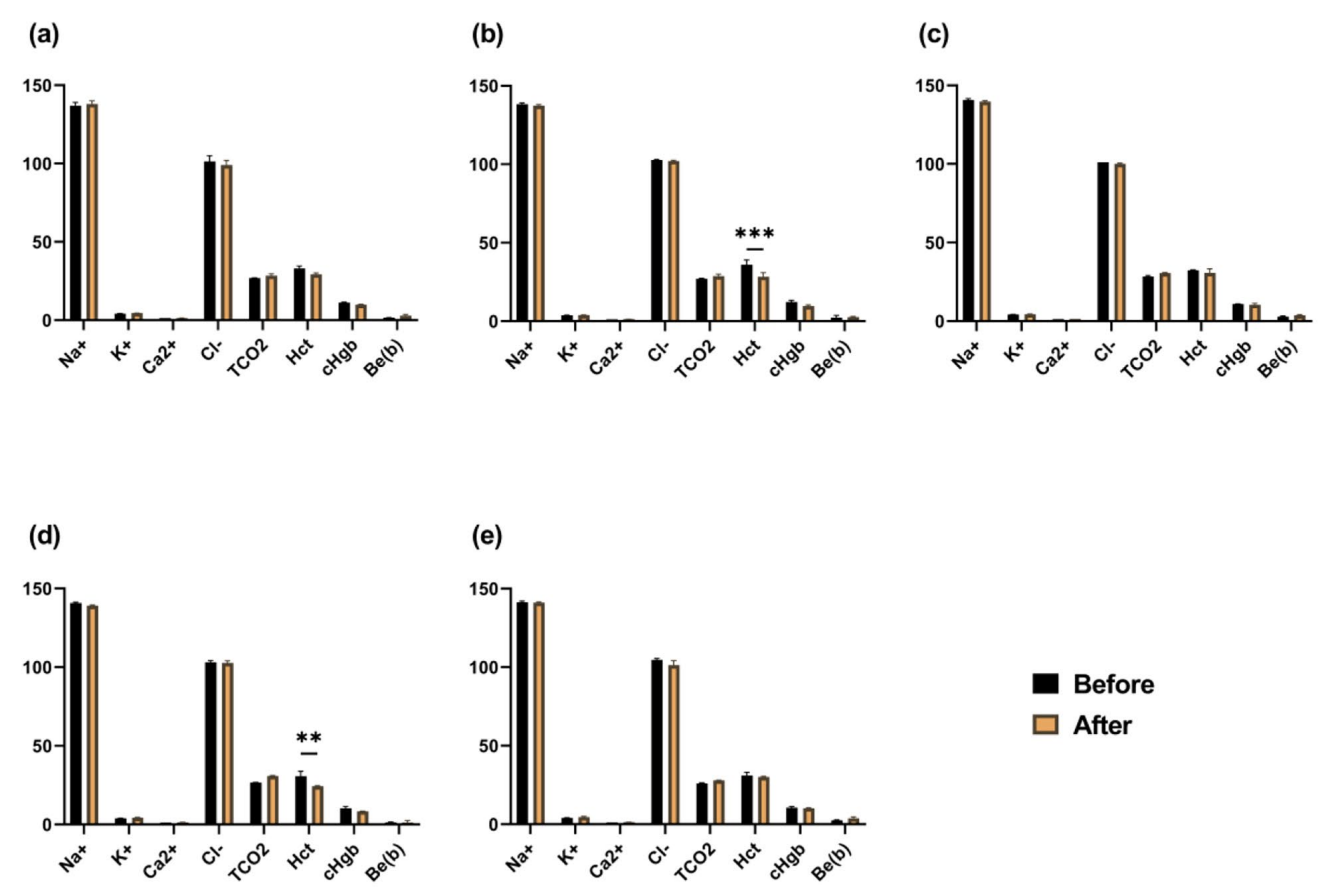
Blood chemistry results before and after fluid administration in Experiment 1. Mean values and SEM for each group are shown Asterisks (*) indicate statistically significant differences (Single asterisk indicates *p* < 0.05; double asterisks indicate *p* < 0.01; triple asterisks indicate *p* < 0.001) between pre- and post-fluid administration values. (**a**): Group1; (**b**): Group2; (**c**) Group3; (**d**) Group4; and (**e**) Control (H/S). (Na^+^, K^+^, Ca^2+^, Cl^−^, TCO2, BE(b) = mmol/L; HCT = %; cHgb = g/dL)

Blood chemistry tests revealed a decrease in Hct levels following the administration of fluids in Groups 2 (NaCl increased) and 4 (most components were increased to match the composition of pig blood) (Fig. 2).

**Fig. 3:**
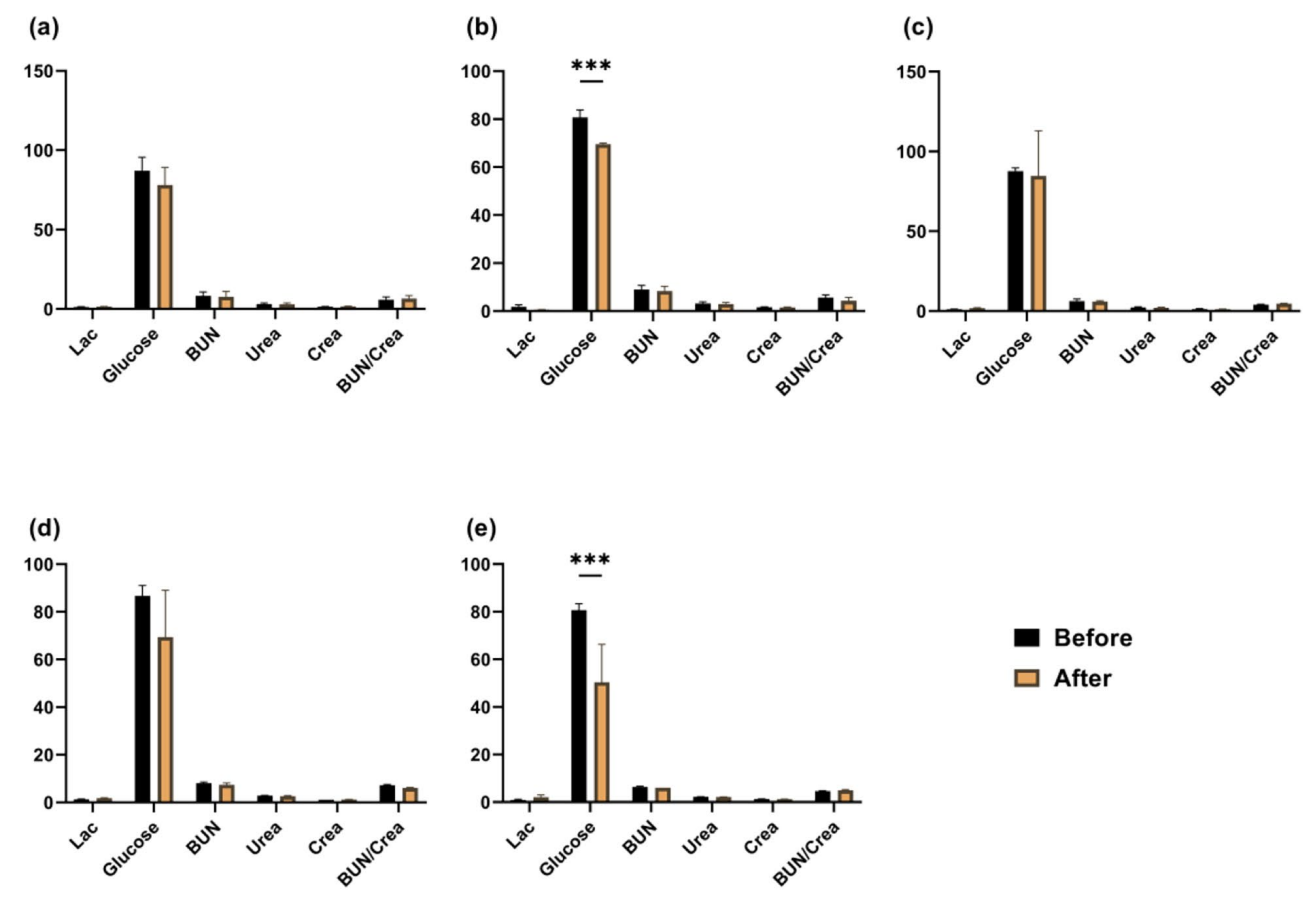
Metabolite levels before and after fluid administration in Experiment 1. Mean values and SEM for each group are presented Asterisks (*) denote statistically significant differences (Triple asterisks indicate *p* < 0.001) between pre- and post-fluid administration values. (**a**): Group1; (**b**): Group2; (**c**) Group3; (**d**) Group4; (and **e**) Control (H/S). (Glu, BUN, Crea = mg/dL; Lac, Urea = mmol/L; BUN/Crea = mg/mg)

Glucose levels exhibited a notable reduction following fluid administration in both Groups 2 and control during metabolite testing (Fig. 3).

Concurrent blood analyses were conducted before and after the injection of fluids to evaluate clinical symptoms and visually observe any negative consequences of fluid administration. No abnormalities were detected and there were no fatalities among the animals.

### Evaluating the efficacy of the customized fluids in diseased pigs

Given the absence of complications in the initial experiment, we selected Group 4 as the fluid for the second experiment to inject into sick pigs. In the second experiment, we analyzed the blood, observed clinical symptoms, and evaluated dehydration. This evaluation was performed before and after fluid administration, as in the first experiment, but was extended until the day following fluid administration.

**Fig. 4:**
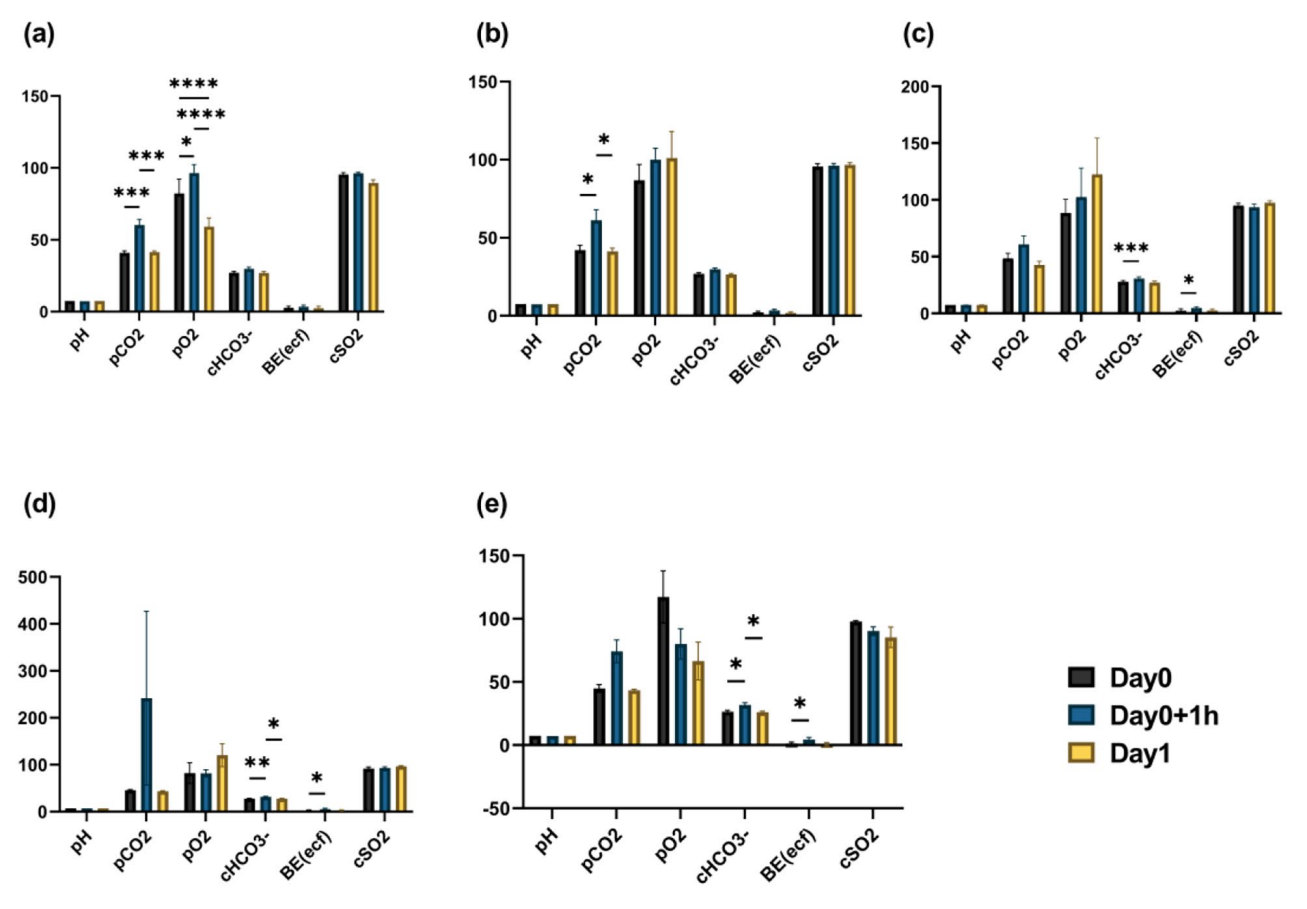
Blood gas analysis results at three time points in Experiment 2. Mean values and SEM for each group are shown. Asterisks (*) indicate statistically significant differences (Single asterisk indicates *p* < 0.05; double asterisks indicate *p* < 0.01; triple asterisks indicate *p* < 0.001; quadruple asterisks indicate *p* < 0.0001) among time points within each group. (**a**): N.C-g; (**b**): N.C-b; (**c**) P.C; (**d**) T1; (**e**) T2. (pCO2, pO2 = mmHg; cHCO3^−^, BE (ecf) = mmol/L; cSO2 = %; N.C-g = healthy pigs without fluids; N.C-b = symptomatic pigs without fluids; P.C = symptomatic pigs with 500mL of H/S; T1 = symptomatic pigs with 500 mL of customized fluids; and T2 = symptomatic pigs with 1 L of customized fluids)

A marginal disparity in blood gas levels was observed between the no-fluid groups (N.C-g, N.C-b) and the fluid groups (H/S, T1, and T2) (Fig. 4). In the group without fluid administration, distinct variations in pCO2 and pO2 levels were observed before and after administration. Within the fluidized bed group, the cHCO3- and BE (ecf) values exhibited a disparity before and after fluid administration.

**Fig. 5:**
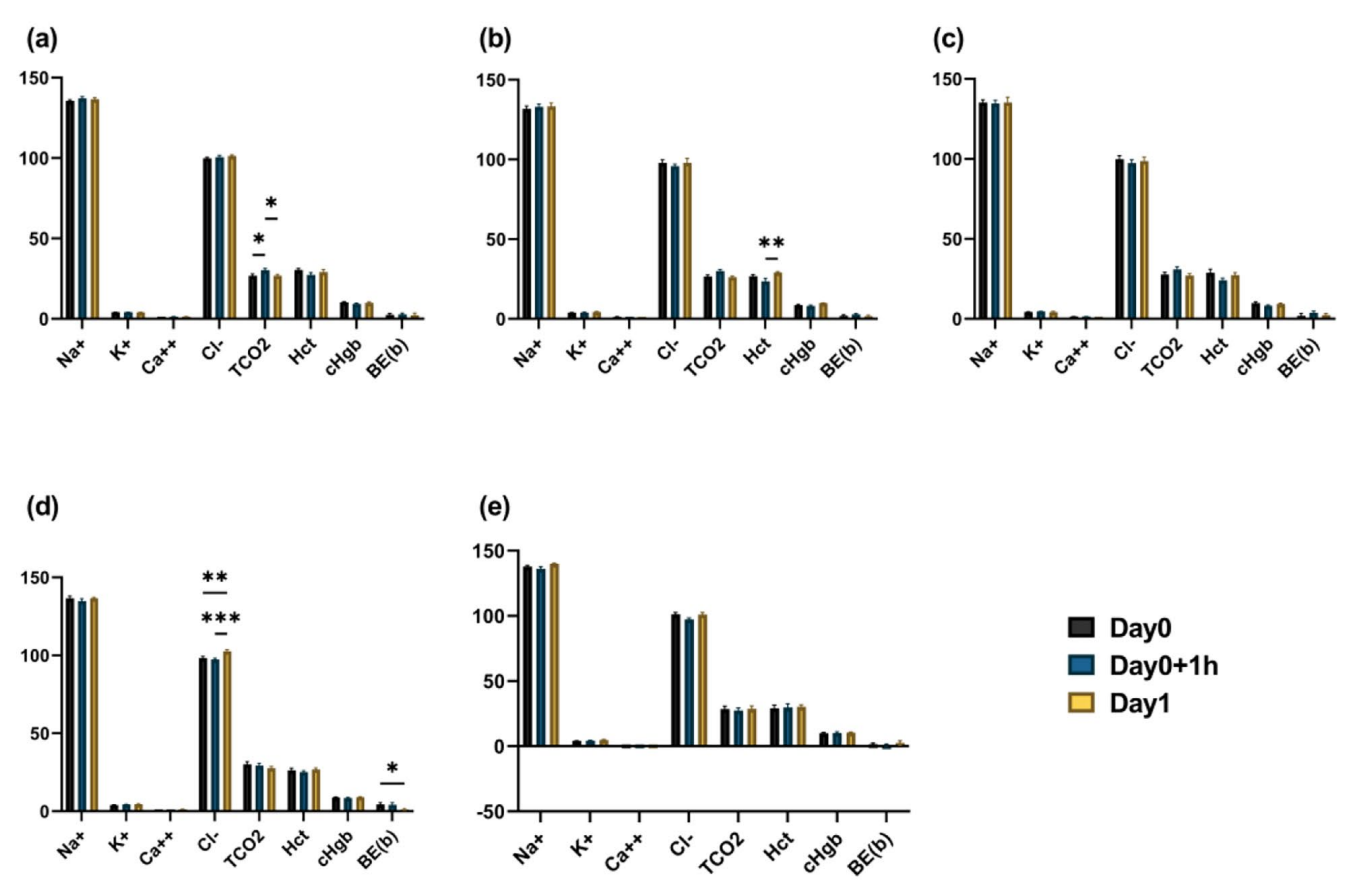
Blood chemistry results at three time points in Experiment 2. Mean values and SEM for each group are presented. Asterisks (*) denote statistically significant differences (Single asterisk indicates *p* < 0.05; double asterisks indicate *p* < 0.01; triple asterisks indicate *p* < 0.001) among time points within each group. (**a**): N.C-g; (**b**): N.C-b; (**c**) P.C; (**d**) T1; (**e**) T2. (Na^+^, K^+^, Ca^2+^, Cl^−^, TCO2, BE(b) = mmol/L; HCT = %; cHgb = g/dL; N.C-g = healthy pigs without fluids; N.C-b = symptomatic pigs without fluids; P.C = symptomatic pigs with 500mL of H/S; T1 = symptomatic pigs with 500 mL of customized fluids; and T2 = symptomatic pigs with 1 L of customized fluids)

Blood chemistry tests revealed that in the control group without fluids (N.C-g), TCO2 levels increased 1 h after fluid administration and reverted to their initial level the following day (Fig. 5). Hct levels decreased and then increased in the symptomatic group that did not receive fluids (N.C-b). After receiving 500 mL of the customized fluids, the group (T1) exhibited elevated Cl- levels one-day post-administration in comparison to the pre-administration levels. Additionally, Be(b) values considerably decreased one day after fluid administration in comparison to the pre-administration levels.

**Fig. 6:**
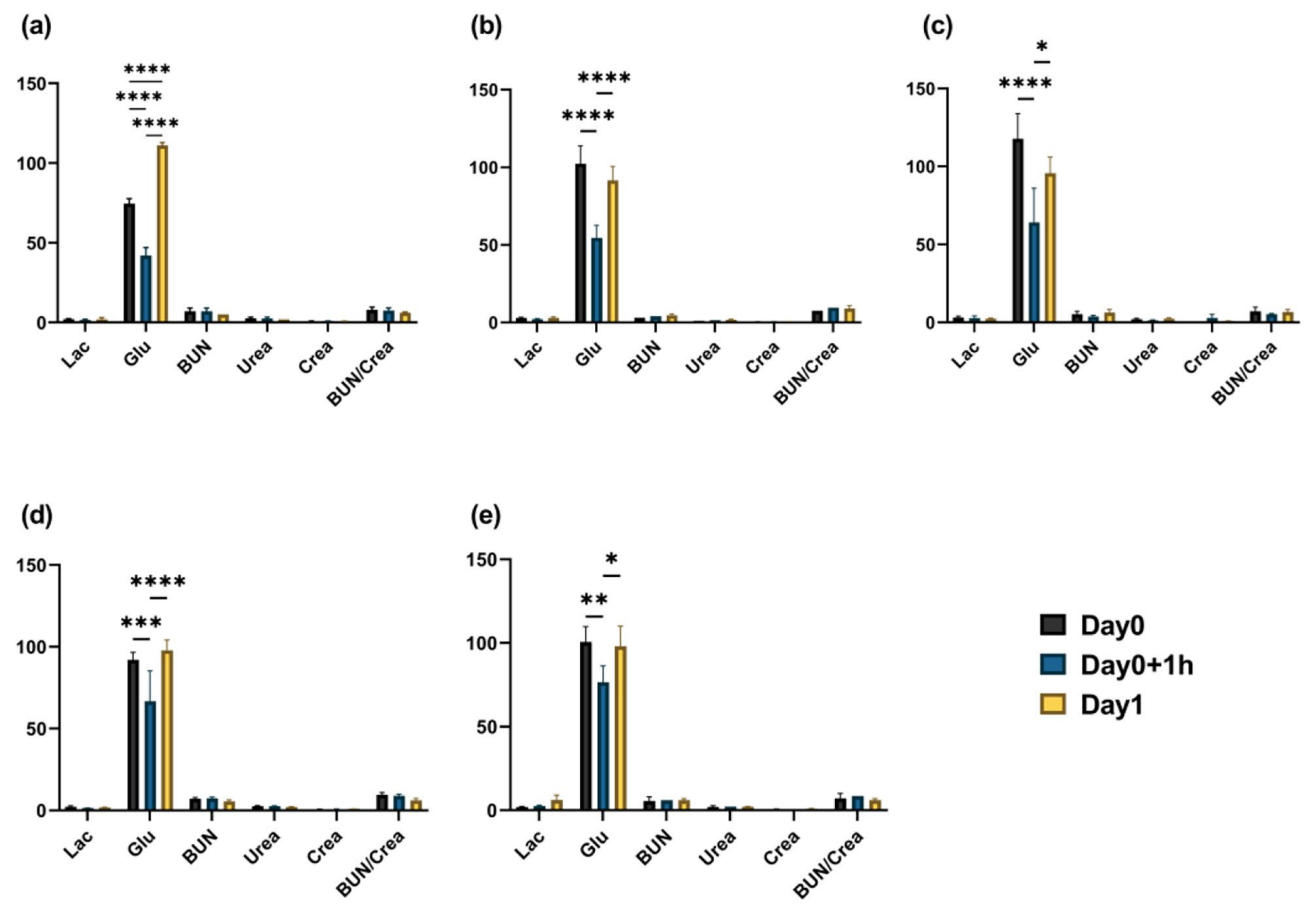
Metabolite levels at three time points in Experiment 2. Mean values and SEM for each group are shown. Asterisks (*) denote statistically significant differences (Single asterisk indicates *p* < 0.05; double asterisks indicate *p* < 0.01; triple asterisks indicate *p* < 0.001; quadruple asterisks indicate *p* < 0.0001) among time points within each group. (**a**): N.C-g; (**b**): N.C-b; (**c**) P.C; (**d**) T1; (**e**) T2. (Glu, BUN, Crea = mg/dL; Lac, Urea = mmol/L; BUN/Crea = mg/mg)

Glucose was the sole metabolite that exhibited a noteworthy difference over time when comparing pre- and post-hydration metabolite levels (Fig. 6). Both the presence and absence of fluids exhibited a consistent declining trend 1 h after fluid administration, followed by restoration to preinjection levels the following day.

No specific clinical symptoms were observed right before or immediately after fluid administration. However, two pigs in the group that received H/S (P.C) died: one on day 1 (Pig A) and another on day 7 (Pig B) after the fluid injection. Additionally, one pig died on day 17 (Pig C) after receiving 1 L of the customized fluid (T2). Pig A displayed signs of rapid deterioration in health, including decreased activity, reduced responsiveness to stimuli, and labored breathing, which were first observed approximately 18 h after fluid administration. Pig B exhibited signs of general deterioration in health, including decreased appetite, lethargy, and weakness, with symptoms first appearing on day 5 after fluid administration. Pig C showed signs of gastrointestinal distress, including intermittent vomiting and diarrhea, which began on day 14 after fluid administration. Dehydration evaluations revealed no enhancement in the dehydration status before or after fluid administration.

## Discussion

A pivotal aspect of fluid therapy in veterinary medicine is the alignment of fluid composition with the animal’s blood composition. H/S is designed to replace fluids and electrolytes in patients with low blood volume or blood pressure; however, its electrolyte composition, although similar, is not identical to that of animal plasma (Table 2). This discrepancy can potentially affect the effectiveness and safety of fluid therapy, especially in critical care situations where precise fluid and electrolyte balance is crucial.

**Table 2:** Ion concentrations compared between Hartmann’s solution and the stable dog, pig, and sepsis pig models

	Na^+^	K^+^	Ca^2+^	Cl^−^
Hartmann’s solution [10]	131	5	2	111
Miniature pig [20]	136.33 ∼ 148.04	2.86 ∼ 6.69	0.96 ∼ 1.62*	95.49 ∼ 106.2
Pig model of severe sepsis [24]	135 ± 5	3.6 ± 0.4	2.2 ± 0.4	103 ± 5

Ion concentrations are expressed in millimoles per litre (mmol/L). For pigs, the reference intervals were written

*Ca^2+^ of pigs was initially presented in mg/dl, but was changed to mmol/L with two decimal places to provide consistency in units

To address this issue, our research focused on creating a customized fluid treatment for pigs that precisely mimics the ionic concentrations present in pig blood. This approach is based on the hypothesis that accurately tailoring the fluid treatment to an animal’s physiological requirements is vital, particularly in instances of shock and substantial fluid depletion, in which the selection and composition of the supplied fluid can exert a considerable impact on the outcomes. The physiology of fluid compartments in animals, such as pigs, is intricate, and the selection of fluid therapy must consider elements such as the distribution of blood volume, the function of various capillaries in fluid exchange, and the body’s general maintenance of water balance [25].

In our first experiment, we wanted to study how changing the amounts of components in H/S affected pigs. All the compounds, except for calcium chloride which has a divalent cation Ca2+, have a monovalent cation paired with a monovalent anion. We chose not to change the amount of calcium chloride because we believed that high calcium levels in the blood could be the most dangerous for pigs compared to other ions. A study demonstrated that acute hypercalcemia, induced by local or systemic calcium administration, had deleterious effects on the pancreas of cats and guinea pigs [26]. The study found that hypercalcemia caused acinar and ductal cell necrosis, intraductal protein precipitates, and eventual pancreatitis in these animals.

We established a specific concentration goal for each ion (Table 1) except for calcium ions. Because of the lack of a reference value for the blood of Jeju Native Pigs (JNPs; *Sus scrofa*), which were utilized as experimental mini-pigs, we established the desired ion concentration by considering the results from blood analysis in other experimental pigs [20, 22, 23]. Subsequently, we computed the ion concentration to generate several groups representing quantitative changes in the number of grams to be added (Table 1). To verify the calculated ion concentrations, we employed inductively coupled plasma mass spectrometry (ICP-MS), a type of ion chromatography, to directly measure the actual ion concentrations. However, specific concentrations of ions such as chloride and lactate were not accessible. Consequently, we conducted our tests by relying on the ion concentrations and quantities of the components in H/S.

Following the production of customized fluids, we measured the pH and osmolarity of each fluid sample. The pH of the solution closely resembled that of H/S (6.0) [27]. However, the osmolarity was significantly greater than that of H/S, ranging from a minimum of 316 moSm/L (Group 1) to a maximum of 346 moSm/L, in contrast to the osmolarity of H/S at 276 moSm/L [27]. The osmolality of the authentic porcine serum was determined by adding the primary electrolyte ions, glucose, and urea. The resulting serum osmolality in pigs was measured to be 284.74 ± 5.73 mEq/L [28]. Hyperosmotic therapy is used to treat intracranial hypertension in patients with traumatic brain injury [29]. However, even with this treatment, it is recommended to keep the osmolality below 320 mOsm/kg H2O, as higher levels may cause heart and immune system problems [30]. In the first experiment, the volume administered (500 mL) was small compared to the body weight (min: 45 kg, max: 75 kg); therefore, no significant clinical signs or symptoms were observed. In a subsequent experiment, Group 4, which had the highest osmolality, was used as the customized fluid. Due to body homeostasis, all relevant values returned to normal on the following day.

In pigs, the ear vein is the most common site for giving intravenous fluids [31]. One study found that ear vein catheters were better than jugular catheters for 7 to 14 days [32]. However, pigs are not used to being restrained like trained animals, which makes placing an IV catheter difficult. Thus, we administered a combined intramuscular injection of Zoletyl and Rompun and maintained respiratory anesthesia with isoflurane.

We used the EPOC^®^ blood analysis system, a point-of-care blood analyzer, to assess its practical use in real-life situations for pigs. One study utilized the simplicity of blood analysis to establish reference intervals for hematological, biochemical, electrolytic, and blood gas parameters in puppies [16].

In the first experiment, the pO2 levels after fluid administration in Group 1 were significantly elevated compared to those before fluid administration (Fig. 1). However, in contrast to previous measurements of blood gas levels, it has been demonstrated that pO2 readings in sheep are most precise when obtained from arterial blood samples [33, 34]. Hence, the alteration in pO2 values seems to lack significance, since it was unaffected by the administration of fluids, but rather influenced by the extraction of venous blood. The pCO2 level, which was anticipated to exhibit an inverse pattern, demonstrated inconsistent outcomes following fluid administration.

In contrast, the Hct levels decreased following the administration of fluids in Groups 2 and 4 (Fig. 2). Hct is the ratio of the volume of red blood cells in the blood and tends to be relatively high when there is a decrease in the amount of water in the body. Notably, HCT levels were found to increase in piglets with diarrhea [35]; therefore, it is likely that this was reduced by fluid administration, and all other groups also showed a reduction in absolute values.

Glucose levels declined following the delivery of fluids in Groups 2 and H/S. The value was markedly below the normal range (89.64–144.9 mg/dL) [36]. Multiple investigations have demonstrated that anesthetics inhibit insulin secretion by obstructing ATP-sensitive K + channels in β-cells, resulting in elevated blood sugar levels (hyperglycemia) [37]. The administration of isoflurane through inhalation and injection of ketamine/xylazine in mice [38] and the use of propofol or pentobarbital in pigs [39] have been demonstrated to elevate glucose levels. The findings of these studies contradict those of the current study, indicating that the administration of fluids in this study may have caused a slight dilution in the blood analyzed shortly after fluid administration, leading to lower glucose levels. Following confirmation that the fluid composition used in the first experiment did not exhibit any adverse effects on blood analysis or clinical symptoms, Group 4, in which most of its components were adjusted to match the pig fluids, was utilized as a customized fluid in the second trial.

In the second experiment, we aimed to determine the efficacy of customized fluids in dehydrated pigs. While some pigs exhibited symptoms of lethargy and vomiting, these clinical signs were not attributed to a specific bacterial or viral infection based on diagnostic testing. However, it is important to note that our diagnostic approach, which relied on standard fecal screening tests, may have had limitations in terms of sensitivity and specificity. There is a possibility that other, less common pathogens could have been involved in causing these symptoms. In future studies, incorporating more comprehensive diagnostic testing, including the use of rapid kits for specific pathogens, could help to better characterize the potential infectious causes of dehydration in our study population.

Despite the limitations of our diagnostic approach, the cause of the observed symptoms was presumed to be related to dehydration or other non-infectious factors, such as diet or environmental stress. These factors were not specifically investigated in our study, as our primary focus was on evaluating the efficacy of our customized fluid therapy. However, exploring these potential non-infectious causes of dehydration could provide valuable insights and should be considered in future research. In contrast to the initial experiment, the second experiment involved conducting blood tests on the day following fluid delivery, while simultaneously evaluating dehydration levels.

Initially, blood gas measurements exhibited distinct pattern variations between the groups without fluid (N.C-g, N.C-b) and the groups with fluid (H/S, T1, T2) (Fig. 4). Within the fluidized group, both pCO2 and pO2 exhibited consistent rather than contrasting patterns. This similarity can be attributed to the factors previously discussed. Conversely, in the fluidized bed group, the cHCO3^−^ and BE (ecf) values exhibited a disparity before and after fluid administration. For cHCO3^−^, both H/S and customized fluids are anticipated to be influenced by lactate. Furthermore, the correlation between the trends of HCO3^−^ and pCO2 is typically examined similarly [40]. In this case, both trends indicated the same pattern, suggesting that respiratory acidosis may have occurred as a result of reduced respiration caused by anesthesia. The BE(ecf) lacks significance because the anion gap, which denotes the disparity between cations and anions, holds greater clinical relevance. Moreover, the base excess in the extracellular fluid (ecf) does not accurately reflect the base excess in the entire body [41]. Thus, in this investigation, neither value exhibited any significant difference compared to the control group, which received H/S despite the administration of customized fluids.

Blood chemistry readings demonstrated that, in the healthy group without fluids (N.C-g), TCO2 increased by 1 h after fluid administration and returned to its original level the next day (Fig. 5). TCO2, similar to pCO2, is an indicator of the overall level of carbon dioxide in the blood. This indicates that the respiratory disturbance caused by anesthesia is evident in the TCO2 levels. During anesthesia in horses, the levels of pCO2, bicarbonate, and tCO2 increased. A previous study revealed that the increase in tCO2 was more pronounced with sevoflurane than with isoflurane [42].

In the group of symptomatic pigs that did not receive fluids, there was an initial decline, followed by an increase in Hct levels. As previously indicated, an increase in Hct level is indicative of dehydration. As no fluids were administered, the Hct levels increased again on the following day. After receiving 500 mL of customized fluid (T1), the group showed slightly elevated Cl^−^ levels one day later compared to the previous day. However, all levels remained within the normal range of 86.8–103.3 mmol/L [36], except for two pigs whose exhibited higher levels (104 mmol/L). A previous study demonstrated hyperchloremia in calves following the administration of hypertonic saline [43]. However, no significant difference was observed in the 1 L group, in which the volume of fluids was higher. Therefore, it is unlikely that this is the underlying reason. Be(b) is a measure of the BE in the blood, which is similar to the BE (ecf) observed in blood gas tests. After fluid administration, the Be (b) value was significantly lower than before, but still fell within the normal range. The absolute change in value was similar in the group receiving H/S, indicating that there was no significant difference with this fluid, at least in terms of the Be(b) value. Glucose levels exhibited a reduction for analogous reasons as in the initial trial, followed by an increase on the subsequent day as a result of homeostasis (Fig. 6).

Along with collecting blood, we assessed the pigs’ clinical symptoms and dehydration. We found that the symptomatic pigs did not show any improvement in their symptoms or dehydration. The deaths of three pigs during our study highlight the importance of considering pre-existing health conditions when administering fluid therapy. Pig A (H/S group)’s rapid deterioration in health, which was first observed approximately 18 h after fluid administration, suggests that the underlying cause was likely a severe, undiagnosed health condition that was not detected during our initial screening process. Similarly, Pig B (H/S group)’s general deterioration in health, with symptoms appearing on day 5 after fluid administration, indicates the presence of a pre-existing health condition that was not identified during the initial screening. Pig C (1 L of the customized fluid group)’s gastrointestinal distress, which started on day 14 after fluid administration, is less likely to be directly related to the fluid therapy, given the extended time between fluid administration and symptom onset. However, without further diagnostic testing, the exact cause of death in this pig cannot be definitively determined.

The delayed onset of symptoms in these pigs, ranging from 18 h to 14 days after fluid administration, strongly suggests that the observed mortality was not a direct result of the fluid therapy. Instead, these cases underscore the importance of thorough screening and monitoring of animals in fluid therapy studies to identify and manage potential pre-existing health conditions [44]. In future studies, we will work to improve our screening methods to better detect and exclude animals with potentially life-threatening health issues.

Overall, the findings indicated that while there were considerable variations in certain values, these discrepancies were primarily attributable to the limited sample size (three to four pigs per group), use of anesthetics during fluid administration, and not the characteristics of the fluids themselves. Hence, the first experiment revealed no detrimental effects on pig health when personalized fluids were used in healthy pigs. Furthermore, the present study found no noteworthy alterations in blood test results after administering personalized fluids compared to H/S in both healthy and symptomatic individuals. This illustrates that customized fluids do not have a detrimental impact on pigs and can serve as a substitute for the conventional H/S. Furthermore, this implies that when commercially accessible fluids are unavailable, it could be advantageous to formulate and provide customized fluids according to the blood analysis outcomes of each individual.

This study had several limitations. First, the limited number of animals per group (3–4) led to certain fluctuations in the blood analysis findings. For instance, in the group in which H/S was administered, one animal died within 24 h of receiving the solution, precluding blood analysis. Nevertheless, current laboratory animal facility constraints have led to the use of a limited number of animals. Future studies with larger sample sizes could also explore the potential for sex-specific responses to fluid treatments by examining male and female pigs independently. This approach would allow for a more comprehensive understanding of how sex might influence the efficacy and safety of customized fluid therapy in pigs. The small sample size in this study also precluded the use of power analysis to determine the optimal number of animals required to detect significant differences between the treatment groups. This limitation may have affected the statistical power of our findings and the ability to draw definitive conclusions. Future studies should employ larger sample sizes based on appropriate power calculations to validate and expand upon our preliminary results. Second, because a portable blood analyzer was utilized to analyze the blood, it is unfavorable. Portable blood analyzers offer the convenience of not requiring sample storage or transportation. However, it is susceptible to environmental factors, such as temperature and humidity, and requires meticulous calibration and validation [45]. In future studies, it is necessary to analyze the blood using conventional laboratory devices. Third, the use of anesthesia could influence the parameters being examined such as pO2, pCO2, glucose [46, 47]. Future studies could consider alternative methods for fluid administration that do not require general anesthesia, such as using a restraint device or sedation, to further minimize the potential confounding effects of anesthesia on the results. Finally, due to the housing conditions of the pigs, it was challenging to simultaneously conduct a comprehensive assessment of dehydration by measuring weight loss, urine output, and other relevant factors along with the blood analysis. This would have yielded a more precise understanding of the improvement in dehydration as the evaluation of dehydration was predominantly based on subjective judgment.

## Conclusion

Our study showed that using customized fluid therapy in pigs, designed to match the ion levels in pig blood, is both possible and safe. We did not see any major negative effects on the pigs’ health when comparing the customized solution to the standard H/S. However, the study was limited by a small sample size and environmental factors affecting the portable blood analyzer used. Subsequent research should prioritize increasing the sample size and utilizing more controlled laboratory conditions for blood analysis. The strategy developed in this study has the potential to offer a practical solution in situations where commercial fluids are not feasible in veterinary medicine, particularly in pigs, by aligning treatments more closely with their physiological requirements. This approach charted a new direction, demonstrating a pivotal shift in veterinary fluid therapy towards more species-specific and situation-adapted interventions.

## Methods

### Experimental design

#### Experiment 1: evaluating the stability of customized fluids in healthy pigs

The objective of this experiment was to assess the safety and potential adverse effects of the customized fluids compared to the standard Hartmann’s solution in healthy pigs. We prepared four different fluid compositions by modifying the concentrations of Na+, K+, Cl-, and lactate in Hartmann’s solution (Table 1). Fifteen healthy pigs were randomly allocated into five groups (*n* = 3 per group), including one control group receiving Hartmann’s solution (H/S) and four treatment groups receiving the customized fluids (G1, G2, G3, and G4). Each pig received a single 500 mL infusion of the assigned fluid over a period of 30 min. Blood samples were collected before and after fluid administration for comparative analysis of blood parameters using the EPOC^®^ blood analysis system. The pigs were closely monitored for any adverse reactions or clinical signs following fluid administration.

#### Experiment 2: assessing the efficacy of customized fluids in pigs with clinical symptoms

The objective of this experiment was to evaluate the effectiveness of the customized fluids in pigs presenting with clinical symptoms such as dehydration, vomiting, and diarrhea, compared to the standard Hartmann’s solution. Based on the results from Experiment 1, we selected the fluid composition from Group 4, which closely mimicked the ionic concentrations in pig blood, as the customized fluid for this experiment. We established five groups (*n* = 4 per group): a healthy control group (N.C-g), a symptomatic control group (N.C-b), a symptomatic treatment group receiving 500 mL of Hartmann’s solution (P.C), and two symptomatic treatment groups receiving either 500 mL (T1) or 1 L (T2) of the customized fluid. The pigs within each group were matched for age and weight. Fluid administration, blood sample collection, and clinical assessments were performed at three time points: before fluid administration, 1 h after fluid administration, and 24 h after fluid administration. Blood samples were analyzed using the EPOC^®^ blood analysis system, and clinical symptoms, including dehydration, were assessed by experienced veterinarians.

### Animals

A total of 35 Jeju Native pigs (JNPs, *Sus scrofa*) were evaluated at Cronex Corporation, Cheongju, Chungcheongbuk-do, Korea. The first experiment involved the selection of three pigs from each group (*n* = 15, Table 3). For the second experiment, four pigs were selected per group (*n* = 20, Table 4). The subjects were randomly selected from the existing herd, and pigs within the same group were similar in terms of age and weight. The symptomatic pigs (*n* = 16) were selected for the second experiment. The term “symptomatic” encompasses not only a decrease in appetite and an increase in lethargy (*n* = 12), but also includes symptoms such as diarrhea (*n* = 3), vomiting (*n* = 1), and other indications that necessitate the administration of fluid treatment to improve dehydration. Each group was segregated into individual enclosures equipped with straw bedding, feed, and drinking water. Before blood collection, the pigs were acclimated to the experimental settings for a duration of one week. They were fed a commercially formulated diet twice daily (Daehanfeed, Korea; Table 5). Each pig was provided with an individual water bowl that was replenished with clean, fresh water twice daily to ensure ad libitum access. The water bowls had a capacity of 4 L, which was sufficient to meet the daily water requirements of growing pigs [48]. The interior temperature and humidity levels were maintained within the range of 22–25 °C and 50–70%, respectively. The pigs underwent daily health and welfare evaluations during which signs of distress or disease were carefully recorded. Healthy pigs displayed uniform average body temperature, physical activity level, and appetite, with no noticeable clinical signs.

**Table 3:** Demographic characteristics of animals in the first experiment

	Sex		Age (months)	Weight (kg)
Hartmann’s solution	Male	2	8	62.5 ± 0.65
	Female	1		
Group 1	Male	0	9	57.75 ± 0.85
	Female	3		
Group 2	Male	3	9	72 ± 0.91
	Female	0		
Group 3	Male	1	7	46.25 ± 0.48
	Female	2		
Group 4	Male	0	6	51 ± 0.70
	Female	3		

Weight is presented as mean ± standard error of the mean (SEM)

**Table 4:** Demographic characteristics of animals in the second experiment

	Sex		Age (months)	Weight (kg)
N.C - g	Male	1	3	16 ± 0.41
(No fluid, good condition)	Female	3		
N.C - b	Male	1	3	12.38 ± 0.69
(No fluid, bad condition)	Female	3		
P.C	Male	2	4	20.75 ± 1.65
(Hartmann’s solution)	Female	2		
T1	Male	3	4	17.25 ± 1.50
(Customized Fluid)	Female	1		
T2	Male	3	4	22.75 ± 2.75
(Customized Fluid 1 L)	Female	1		

Weight is presented as mean ± standard error of the mean (SEM)

**Table 5:** Chemical composition of commercially formulated diet

Component	Amount	Component	Amount
Crude Protein (min.)	17.00%	Phosphorus (P) (min.)	0.65%
Lysine (min.)	1.00%	Salt (NaCl) (min.)	0.32%
Crude Fat (min.)	3.20%	Salt (NaCl) (max.)	0.82%
Crude Fiber (max.)	7.00%	Zinc (Zn) (min.)	175ppm
Calcium (Ca) (min.)	0.50%	Selenium (Se) (min.)	0.30ppm
Calcium (Ca) (max.)	1.00%	Phytase (A.Oryzae) (min.)	227 FYT/lb

### Developing fluid samples with adjusted composition

#### Conducting investigations on the components of customized fluid

H/S (JW Pharmaceutical, Seoul, Korea) was used as the foundational solution and the concentration of each ion was modified by changing the quantity of each constituent. H/S consists of four components: sodium chloride (molecular weight [MW]:54.4), sodium lactate (MW:112.06), potassium chloride (MW:74.55), and calcium chloride dihydrate (MW:147.01). Four groups were created: in Group 1 the amount of sodium lactate was increased. In group 2 the amount of NaCl was increased. In group 3, the amount of KCl was increased. In Group 4 the amounts of NaCl, KCl, and sodium lactate were increased to closely match the fluid composition to that of pig blood.

#### Producing tangible fluid samples

**Fig. 7:**
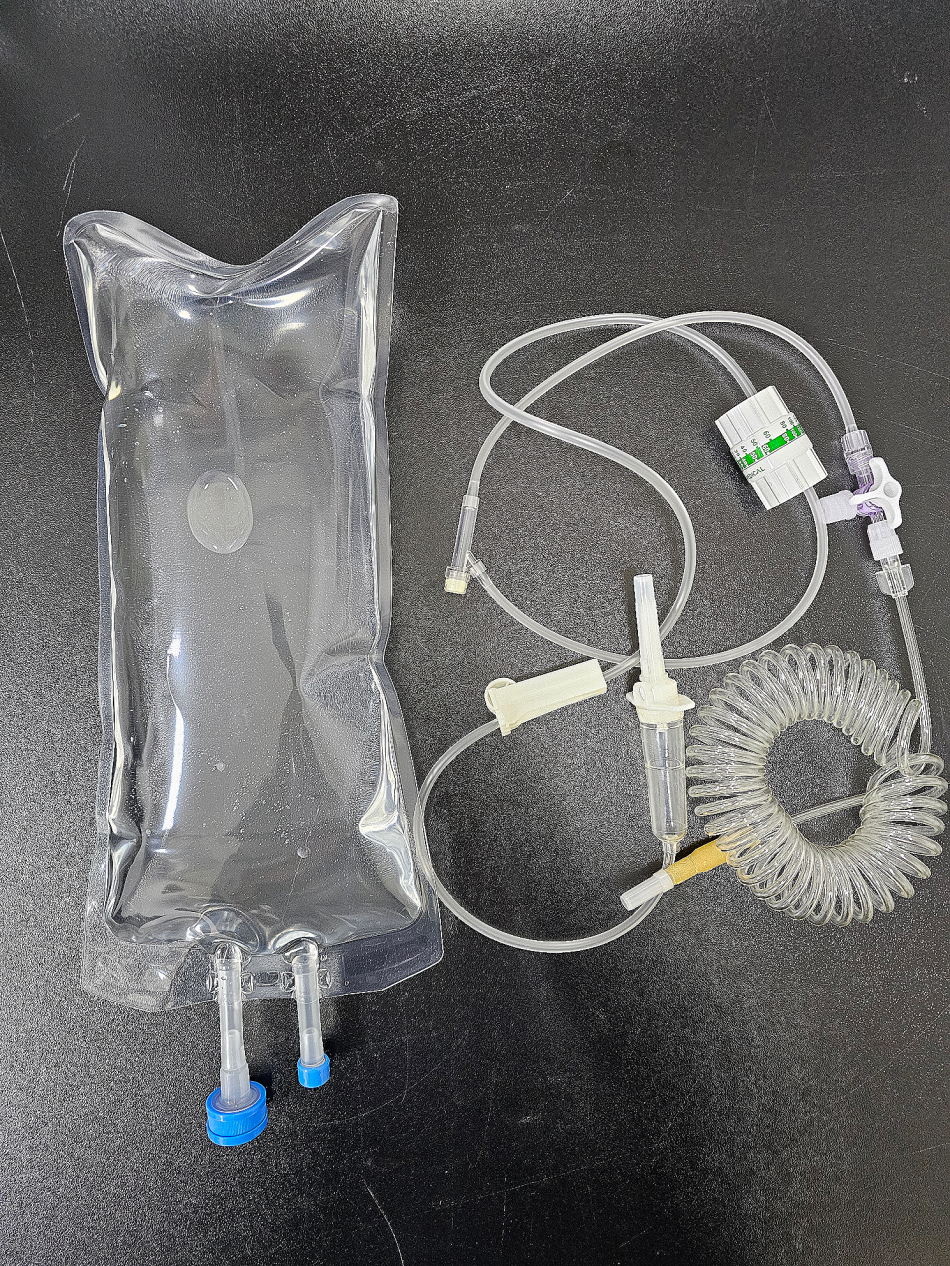
A sample of an infusion pack and a recently designed infusion set (Sungwon Medical, Korea) for this experiment

Fluid samples were prepared to match the number of participants in each group. An IV solution pack consists of three essential elements: a fluid bag containing the fluid, a port for injecting the fluid, and a port for administering the fluid. The fluid bags are composed of polypropylene (PP) and a non-polyvinyl chloride (non-PVC) polymer. Fluid bags and all other components were purchased from Isupply (Seongnam, Korea). To minimize contamination, we autoclaved each port and a 1 L glass bottle (DURAN, Germany). The fluid bag was sterilized using a low-temperature plasma sterilizer (MAXterileTM PS60; DAIHAN Labtech, Korea). Subsequently, the compounds were blended to achieve the same composition as the group and transferred to 1 L containers. Following sterilization, the medication administration port was sealed initially, and the fluid prepared in the fluid bag was introduced using a 50 mL syringe (Fig. 7). The fluid administration port was subsequently sealed and placed in a refrigerator at a temperature of 4 °C. Before the experiment, the samples were removed from the refrigerator and allowed to reach room temperature (22–25 °C) for 3 h.

### Anesthesia and injection of customized fluid

All animals underwent a physical pre-anesthetic examination and an overnight fasting period, during which they were only allowed to consume water for drinking. Before administering customized fluids, all pigs in each group were anesthetized and manually restrained to ensure fluid delivery. The induction procedure involved administering a mixture of 17.5 mg/kg Zoletyl (an anesthetic including Tiletamine and Zolazepam, Virbac, France) and 25 mg/kg Rompun (an analgesic containing Xylazine and Methylparaben, Elanco, USA) through an intramuscular (IM) injection. All the pigs in each group quickly became sedated within three to four minutes and they all became laterally recumbent without showing any signs of excitement. Following the insertion of an intravenous (IV) catheter into the auricular vein, the pig underwent endotracheal tube (ETT) intubation with ventral recumbency, and anesthesia was sustained using isoflurane (Isotroy, Troikaa Pharmaceuticals, India) at an inhaled concentration of 1.5–1.8% in oxygen. Anesthesia depth was evaluated at 10-minute intervals by assessing muscle relaxation, absence of movements, of the jaw tone, and absence of palpebral reflex to ensure that the animals were unconscious. Throughout the procedure, physiological data including heart rate (HR), blood pressure (BP), respiratory rate (RR), body temperature (BT), mucous membrane color, capillary refill time (CRT), and pCO2 were continuously monitored and recorded.

In the initial trial, 500 mL of fluid was administered to all healthy pigs. In the subsequent trial, the pigs that received fluids were administered 1 L of fluid in addition to the initial 500 mL. Intravenous fluid was delivered to the pigs at an average rate of 20 ml/kg BW per hour, with adjustments based on each individual’s weight and medical condition. Following the completion of the fluid injection, the pigs’ vital signs, including BT, RR, HR, and CRT were monitored at 15-minute intervals until the animals were able to keep themselves in a voluntarily sternal recumbent position. To prevent hypothermia, the animals were kept warm during recovery using a heating pad and microwavable gel pack.

### Blood collection and analysis

Blood samples were collected from the external jugular vein using a 5-mL syringe (Korean Vaccine Co., Seoul, Korea). They were collected at consistent daily intervals for each group by a single staff member who was blinded to the group allocation of the animals. The groups were randomly assigned labels in alphabets such as A, B, C, and D, which did not correspond to the group names used above. Immediately after collection, each blood sample was analyzed individually using the EPOC^®^ blood analysis system (Woodley Equipment Company Ltd, Lancashire, UK) within 5 min. The following parameters were evaluated: pH; partial pressure of carbon dioxide (pCO2); partial pressure of oxygen (pO2); base excess of extracellular fluid (BE ecf); the concentration of bicarbonate (cHCO3); oxygen saturation (cSO2); total carbon dioxide (tCO2); levels of sodium, potassium, calcium, and chloride; hematocrit (Hct); the concentration of circulating hemoglobin (cHgb); glucose and lactate concentrations; blood urea nitrogen (BUN); urea nitrogen (Urea); creatinine (Crea); and the ratio of BUN/Crea. In the first experiment, blood sampling and analysis were conducted on two occasions: before fluid administration and 1 h after fluid administration. In the second experiment, blood sampling and analysis were performed three times, including before fluid administration and 1 h and 24 h after fluid administration. This was performed to mitigate the effect of anesthesia.

### Assessment of clinical signs and dehydration

Each pig was examined for clinical symptoms and dehydration during blood sampling and subsequent analyses. The observed clinical symptoms included body temperature, heart rate, respiratory rate, and behavior. Dehydration was assessed by examining skin turgor, ocular appearance, mucous membrane condition, and capillary refill time. Skin turgor was assessed by elevating the skin on the posterior aspect of the neck and noting the time taken for it to revert to its normal state. The ocular appearance was evaluated based on the extent of concavity and desiccation. The condition of the mucous membrane was assessed based on the color, wetness, and texture of the gums. Capillary refill time was assessed by applying pressure to the gum and measuring the duration until the usual color was restored.

### Euthanasia

The euthanasia process followed the AVMA guidelines for euthanizing animals (2020 edition) [49]. At the end of all experiments, euthanasia was performed after Rompun overdose, via intramuscular injection of succinylcholine at a dosage of 1 mL/10 kg. Subsequently, the bodies were cryogenically preserved at -20 °C and placed under the care of a mortuary specializing in animal carcass disposal for later disposal.

### Statistical analysis

Data were analyzed using GraphPad Prism (version 9.3, GraphPad Software Inc., San Diego, CA, USA). Prior to conducting the analysis of variance (ANOVA), the normality of the data was determined using the Shapiro-Wilk test, and the homogeneity of variances was examined with Levene’s test. These initial assessments guarantee that the ANOVA’s assumptions are satisfied. One-way ANOVA with Tukey’s multiple comparisons test and 2-way ANOVA with Šídák’s multiple comparisons test were used to determine the statistical differences between parameters. If the values were missing, a mixed-effects model was used. Following the second experiment, one of the four pigs that received H/S died within 24 h of receiving the fluid. Consequently, the blood analysis data from this particular pig were omitted from the study because of its unmeasurable status. The significance level was set at *p* < 0.05. The results are presented as mean ± standard error of the mean (SEM).

## Data Availability

The datasets used and/or analyzed during the current study are available from the corresponding author on reasonable request.

## Abbreviations

pCO2: Partial pressure of carbon dioxide (pCO2)
pO2: Partial pressure of oxygen
BE ecf: Base excess of extracellular fluid
cHCO3: The concentration of bicarbonate
cSO2: Oxygen saturation
tCO2: Total carbon dioxide
Hct: Hematocrit
cHgb: The concentration of haemoglobin
Glu: Glucose
Lac: Lactate
BUN: Blood urea nitrogen
Urea: Urea nitrogen
Crea: Creatinine
